# The small RNA complement of adult *Schistosoma haematobium*

**DOI:** 10.1371/journal.pntd.0006535

**Published:** 2018-05-29

**Authors:** Andreas J. Stroehlein, Neil D. Young, Pasi K. Korhonen, Ross S. Hall, Aaron R. Jex, Bonnie L. Webster, David Rollinson, Paul J. Brindley, Robin B. Gasser

**Affiliations:** 1 Melbourne Veterinary School, Department of Veterinary Biosciences, Faculty of Veterinary and Agricultural Sciences, The University of Melbourne, Parkville, Victoria, Australia; 2 Population Health and Immunity, Walter and Eliza Hall Institute of Medical Research, Parkville, Victoria, Australia; 3 Parasites and Vectors Division, The Natural History Museum, London, United Kingdom; 4 School of Medicine & Health Sciences, Department of Microbiology, Immunology & Tropical Medicine, The George Washington University, Washington, DC, United States of America; James Cook University, AUSTRALIA

## Abstract

**Background:**

Blood flukes of the genus *Schistosoma* cause schistosomiasis—a neglected tropical disease (NTD) that affects more than 200 million people worldwide. Studies of schistosome genomes have improved our understanding of the molecular biology of flatworms, but most of them have focused largely on protein-coding genes. Small non-coding RNAs (sncRNAs) have been explored in selected schistosome species and are suggested to play essential roles in the post-transcriptional regulation of genes, and in modulating flatworm-host interactions. However, genome-wide small RNA data are currently lacking for key schistosomes including *Schistosoma haematobium*—the causative agent of urogenital schistosomiasis of humans.

**Methodology:**

MicroRNAs (miRNAs) and other sncRNAs of male and female adults of *S*. *haematobium* and small RNA transcription levels were explored by deep sequencing, genome mapping and detailed bioinformatic analyses.

**Principal findings:**

In total, 89 transcribed miRNAs were identified in *S*. *haematobium*—a similar complement to those reported for the congeners *S*. *mansoni* and *S*. *japonicum*. Of these miRNAs, 34 were novel, with no homologs in other schistosomes. Most miRNAs (*n* = 64) exhibited sex-biased transcription, suggestive of roles in sexual differentiation, pairing of adult worms and reproductive processes. Of the sncRNAs that were not miRNAs, some related to the spliceosome (*n* = 21), biogenesis of other RNAs (*n* = 3) or ribozyme functions (*n* = 16), whereas most others (*n* = 3798) were novel (‘orphans’) with unknown functions.

**Conclusions:**

This study provides the first genome-wide sncRNA resource for *S*. *haematobium*, extending earlier studies of schistosomes. The present work should facilitate the future curation and experimental validation of sncRNA functions in schistosomes to enhance our understanding of post-transcriptional gene regulation and of the roles that sncRNAs play in schistosome reproduction, development and parasite-host cross-talk.

## Author summary

Human schistosomiasis is a chronic, neglected tropical disease (NTD) that is predominantly caused by the blood flukes *Schistosoma haematobium*, *S*. *mansoni* and *S*. *japonicum*. Infections by *S*. *haematobium* and/or *S*. *mansoni* are highly prevalent in Africa, affecting ~ 200 million people. The decoding of schistosome draft genomes has, to some extent, improved our understanding of the molecular biology of these parasites and now allows for non-protein-coding regions in these genomes to be characterised. Here, we explored small RNAs in adult *S*. *haematobium* by deep sequencing, reference genome mapping and detailed bioinformatic analyses. This study provides the first genome-wide miRNA and sncRNA resource for *S*. *haematobium*, extending earlier work on schistosomes and facilitating future curation efforts and functional investigations of schistosome sncRNAs. These efforts should enable a better understanding of post-transcriptional RNA modifications, gene regulation and novel aspects of parasite development, parasite-host cross-talk and disease at the molecular level.

## Introduction

Human schistosomiasis is a chronic, snail-borne, neglected tropical disease (NTD) caused predominantly by infections with the blood flukes *Schistosoma haematobium*, *S*. *mansoni* and *S*. *japonicum* [1]. This disease is highly prevalent in sub-Saharan Africa, where infections with *S*. *haematobium* and *S*. *mansoni* affect ~ 200 million people [2]. The decoding of schistosome draft genomes [3–5] has, to some extent, led to an improved understanding of the molecular biology of these parasites and associated disease, but investigations have mostly focused on the protein-coding gene complement. Only recently, non-protein-coding regions in flatworm genomes have come under the spotlight. For instance, small non-coding RNAs (sncRNAs) present in exosomes have been proposed to play a role in modulating parasite-host interplay [6, 7], and the identification of other sncRNA elements suggests similar functionalities in flatworms [6–10]. Clearly, a comprehensive characterisation of sncRNA elements in *S*. *haematobium* and other flatworm taxa, for which data are currently lacking, will likely underpin investigations to understand mechanisms governing parasite-host interactions and disease.

Deep sequencing of the RNA populations of schistosomes has revealed a diversity of non-protein-coding RNA families, including degradation-like small RNA fragments originating from microRNAs (miRNAs) [10–13], small interfering RNAs (siRNAs) and transfer RNAs (tRNAs) [13, 14]. In these studies, miRNAs have been a focus, mostly due to the recognised structures of miRNA precursors and their abundance in the small RNA (sRNA) libraries sequenced to date. Comparative studies have identified that the complements of miRNAs in parasitic flatworms display substantial differences in size and sequence when compared with those of other eukaryotes studied to date [8], but such studies require further support by the sequencing of transcribed sRNA from additional species. To date, most studies have focused on either *in silico* predictions of miRNA precursors from genomic data [11] or on the detection of mature miRNA elements within sequenced sRNAs and the subsequent identification of precursors within genomic data [10].

Parasitic flatworms lack the canonical Piwi pathway [15, 16], suggesting that, whilst many non-coding elements are relatively conserved among eukaryotic species, little is known about the precise composition of non-coding RNA families and their functions in platyhelminths. Due to an absence of sRNA datasets for most lophotrochozoans and the evolutionary distance between lophotrochozoans and model eukaryotes (e.g., *Caenorhabditis* and *Drosophila*) with well-characterised sRNA complements [17–20], efforts to characterise sRNA fragments other than miRNAs in flatworms have been restricted largely to *S*. *japonicum* [13, 14, 21], and are still far from comprehensive.

In the present study, we explored *S*. *haematobium* sRNAs at the transcriptome level. As a foundation for annotating these sRNAs, we improved the annotation of genes, including 5’- and 3’-untranslated regions (UTRs) and tRNA genes. We produced sRNA fragment libraries from adult males and females of *S*. *haematobium*, in order to define conserved miRNAs among schistosomes species for which miRNA data are available, and characterised novel RNA fragments to provide additional evidence for conserved roles of coding and non-coding RNAs in schistosome development and reproduction.

## Methods

### Parasite materials

The sRNA libraries were constructed from total RNA extracted from the same pools of adult male (*n* = 50) and adult female (*n* = 50) *S*. *haematobium* worms used previously for mRNA sequencing [5]. Briefly, this *S*. *haematobium* strain originates from Egypt and was maintained in the Biomedical Research Institute, Rockville, Maryland [22] in *Bulinus truncatus* (snail intermediate host) and *Mesocricetus auratus* (hamster; mammalian definitive host). Helminth-free hamsters were each infected with 1000 cercariae. After 90 days, paired male and female adults of *S*. *haematobium* were collected from *M*. *auratus*, following the perfusion of the mesenteric and intestinal vessels using physiological saline (37°C) [5].

### Curation of the *S*. *haematobium* genome annotation

As 5’- and 3’-UTRs were not defined in the *S*. *haematobium* genome [5], they were predicted here by *de novo* assembly of published RNAseq data for adult and egg stages of *S*. *haematobium* (NCBI SRA accession numbers: SRR6655497, SRR6655495 and SRR6655493) using Trinity (release 10 Nov. 2013 [23]). First, transcripts (BioProject: PRJNA431931) were aligned to published genome scaffolds (BioProject: PRJNA78265) using BLAT v. 34x12 [24], with the highest-scoring transcript alignments recorded in GTF format using a Perl script (available from https://github.com/vikas0633/perl/blob/master/blat2gff.pl). Second, combined RNAseq datasets (accession numbers: SRR6655497, SRR6655495 and SRR6655493) were mapped to the genome using Tophat v. 2.1.1 and Cufflinks v. 2.2.1 [25], providing published gene predictions (-G option) [5]. Third, the published gene set (GFF format), gene models of curated gene families (GFF format) [26, 27], and aligned and mapped transcripts (GTF format) were merged using Cuffmerge [25]. Fourth, predicted genes nested within or partially overlapping with another gene were identified using gffread (-E option; https://github.com/gpertea/gffread) and manually curated or removed (redundant gene loci). Finally, the merged gene annotation file was processed using GAG v.2.0.1 (https://genomeannotation.github.io/GAG/), Sequin (https://www.ncbi.nlm.nih.gov/Sequin/) and tbl2asn (https://www.ncbi.nlm.nih.gov/genbank/tbl2asn2/) to confirm high-quality gene models and to remove overlapping, low complexity genes. The gene set, including annotations of the mRNAs, coding domains (open reading frames, ORFs) and UTRs, was submitted to NCBI (BioProject: PRJNA78265) and used in the sRNA analyses described herein. In addition to coding domains, tRNA genes encoded in the genome were predicted using tRNA-scan v.1.4 [28] and manually curated. The identification, classification and positions of repeats in the *S*. *haematobium* genome had been established previously [5].

### Sequencing of sRNAs and quality control

Methods used for the isolation and quality assessment of total RNA were described previously [5]. The sRNA libraries representing male or female adults of *S*. *haematobium* (accession numbers: SRR6655496 and SRR6655494) were constructed (TruSeq Small RNA Library, Illumina) and sequenced (HiSeq 2500 sequencing platform, Illumina) according to manufacturer’s instructions. Reads were filtered for low quality (Phred score: < 35) and adapters removed using Trimmomatic [29], retaining only reads with the 3’-adapter plus ≥ 8 bases.

### Prediction, classification and transcriptional quantification of sncRNAs

For the prediction of miRNAs and the full sncRNA complement, miRDeep2 v. 2.0.0.5 [30] and ShortStack v. 1.2.4 [31] were used, respectively, employing combined (i.e. representing adult male and female *S*. *haematobium*) sRNA sequence libraries. For miRDeep2, known miRNAs of *S*. *mansoni* [10–12, 32] and of *S*. *japonicum* [13, 14, 33, 34], and the Rfam microRNA database (version 11 [35]), which represents members of Lophotrochozoa, were employed. For ShortStack, the minimum read depth was set at 10, and the minimum and maximum Dicer processing sizes at 18 and 30, respectively. Only gene regions containing sRNA read clusters (i.e. precursor elements) predicted to be miRNAs (using miRDeep2) or containing hairpin (HP) structures (using ShortStack) were retained for further analysis. Candidate miRNAs were filtered based on miRDeep2 scores of ≥ 10, or 100% sequence identity to known miRNAs (across the seed region) from other lophotrochozoans represented in miRBase [36] or Rfam [35], or to miRNAs of *S*. *mansoni* and/or *S*. *japonicum* [10–14, 32–34].

Stable (i.e. non-randomly degraded), *bona fide* sRNAs were identified by selecting locus-specific ShortStack sRNA clusters that were less than 20 nucleotides in length and were represented by 100 or more mapped sRNA reads. In addition, ShortStack precursors exhibiting a Watson-Crick strand bias [37] between 0.2 and 0.8 were excluded. Since sRNA sequencing libraries were constructed from 50 male and 50 female worms, the most prevalent sRNA read was selected from each ShortStack precursor to represent the non-redundant consensus sRNA within the precursor (= representative sRNA). Following definition of the non-redundant sRNA sequences, each ShortStack precursor representative sRNA was classified using Infernal v.1.1.1 [38] and assessed for homologs in the Rfam database. ShortStack precursors predicted by Infernal to be tRNA or rRNA were removed from this sRNA set and recorded separately. Subsequently, sRNA clusters that were located entirely within a repeat region were identified. A cluster was defined as repeat-derived or repeat-complementary, depending on whether it was predicted on the same or the opposite strand as the repeat. The stability of selected sRNAs was assessed using the RNAfold software in the ViennaRNA v.2.1.8 package [39]. Transcription levels of miRNAs and all sncRNAs were inferred and normalised as counts per million reads mapped (CPM) using miRDeep2 and ShortStack, respectively.

### Prediction of miRNA binding sites

Each 3'-UTR was screened for miRNA binding sites using the programs miRanda v.3.3a [40] and PITA v.6 [41]. A binding site was considered as valid if it had a score of > 300 (miRanda) and < -10 (PITA).

## Results

### An improved *S*. *haematobium* gene set

The improved gene set for *S*. *haematobium* inferred here contains 10,837 genes coding for 11,140 proteins, compared with 13,073 originally reported, for which no transcript isoforms had been predicted [5]. Of the 10,837 improved gene models predicted here, 4100 (38%) included annotations for both 5’- and 3’-UTRs, 1244 (11%) for 5’-UTRs, and 2596 (24%) for 3’-UTRs.

### High-quality sRNA read sets

**Fig 1:**
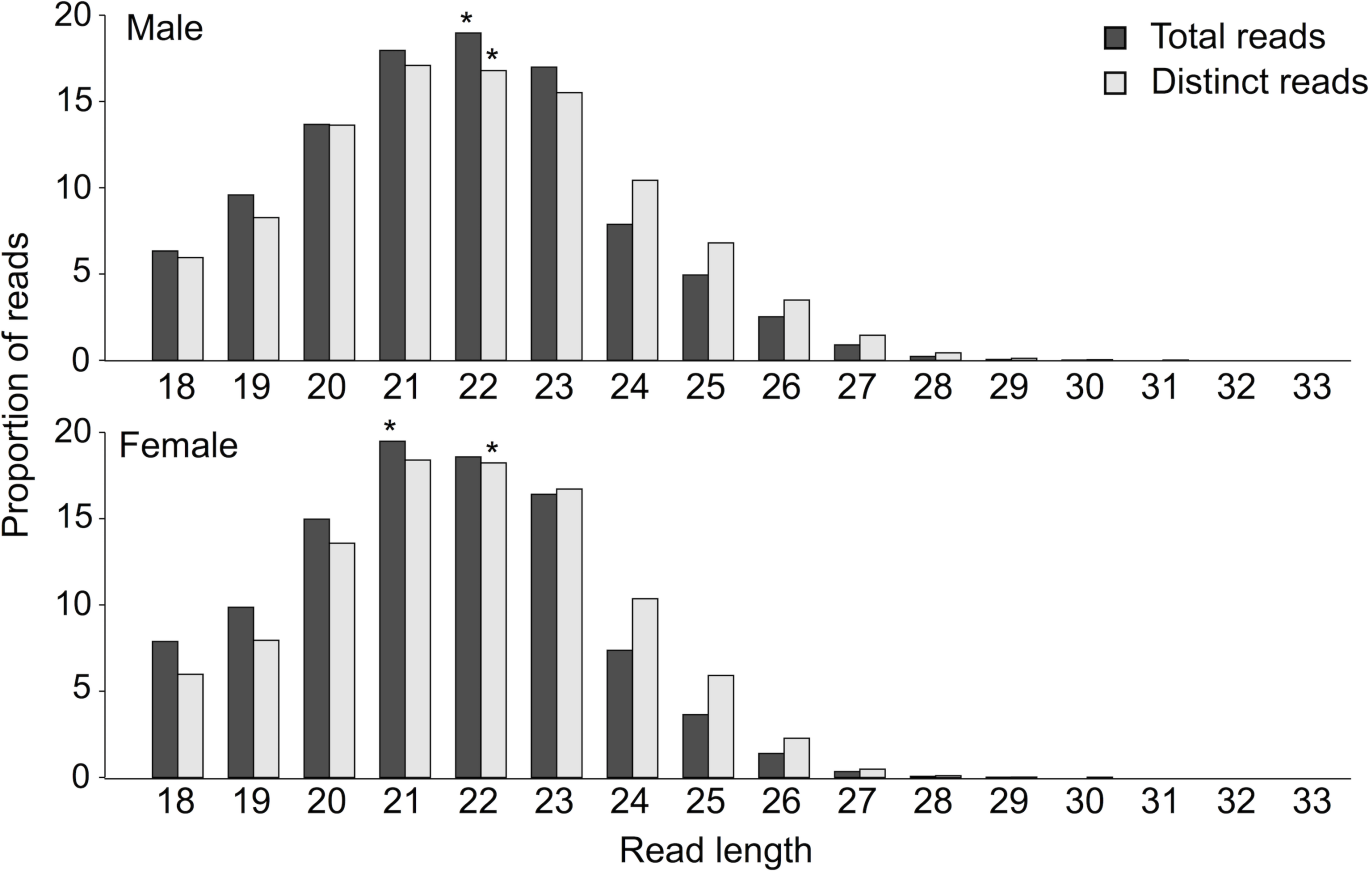
Frequency of read lengths of total and distinct (i.e. after merging redundant reads), quality-filtered small RNA reads representing adult male and female *Schistosoma haematobium*. The median read length for each library is indicated with an asterisk.

In total, ~ 49.6 million and ~ 41.3 million sRNA reads were sequenced for male and female adults of *S*. *haematobium*, respectively (Table 1). Following filtering, ~ 42.2 million (male) and ~ 33.9 million (female) sRNA reads that had a 3’-adapter, lacked 5’-contaminants and were > 18 nucleotides in length were used for analyses (Table 1). Of these, 3,078,078 (7.3%; male) and 3,983,507 (11.8%; female) were distinct (non-redundant) reads (Table 1), with lengths usually ranging between 18 and 28 nucleotides (median of 20 to 21; Fig 1). Most quality-filtered reads from female (68.1%) and male worms (80%) mapped to the *S*. *haematobium* genome and were then used to identify and classify sncRNA elements transcribed in adult *S*. *haematobium*.

**Table 1:** Characteristics of small RNA libraries established from male and female adults of *Schistosoma haematobium*.

Raw data	Male	Female
Total reads	49,683,793	41,390,766
Clean reads^a^	42,165,608 (84.9%)	33,903,550 (81.9%)
Distinct reads^b^	3,078,078 (7.3%)	3,983,507 (11.8%)
Mapped reads^c^	33,723,158 (80.0%)	23,070,632 (68.1%)

^a^ Reads that retained a 3’ adapter, lacked a 5’ contaminant and were greater than 18 nucleotides in length.

^b^ Non-redundant (i.e. merged) set of small RNA reads sequenced

^c^ Mapped to the reference *S*. *haematobium* genome

### *S*. *haematobium* miRNAs

**Fig 2:**
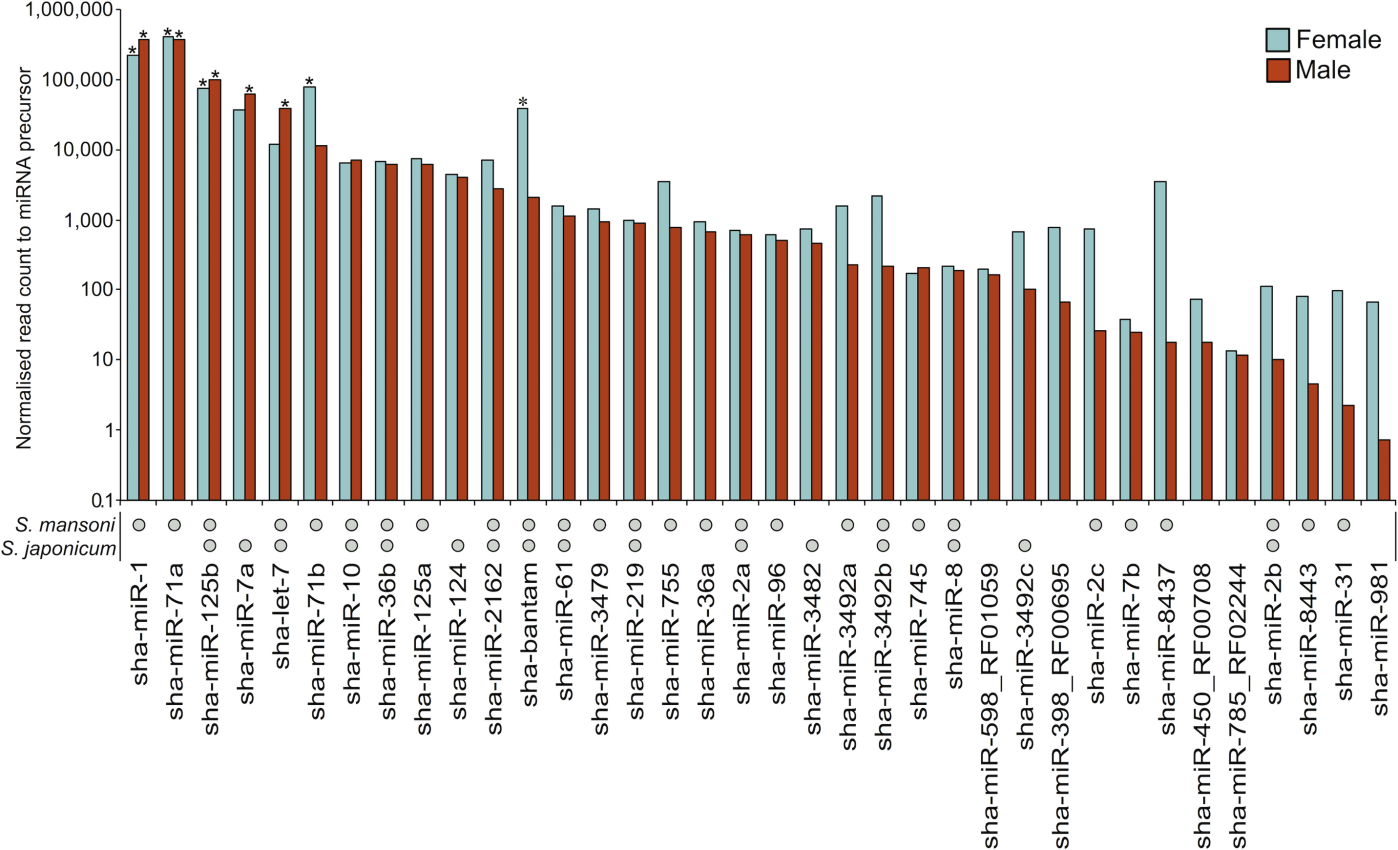
The microRNAs (miRNAs) transcribed in male and female adults of *Schistosoma haematobium* that are homologous to known miRNA seeds, including those of *S*. *mansoni* and *S*. *japonicum*. The miRNAs are ordered from highest to lowest transcription in the male adult, based on normalised read counts. Asterisks denote the five most highly transcribed miRNAs in the male and female adults; circles indicate miRNAs with homologous seeds in other schistosome species.

A total of 89 transcribed miRNAs were identified in *S*. *haematobium*, which compares with 112 in *S*. *mansoni* [10] and 78 in *S*. *japonicum* [13]. Of these 89 miRNAs, 27, 16 and 12 miRNAs were inferred to have an homologous miRNA seed in *S*. *mansoni*, *S*. *japonicum* and among the three schistosome species, respectively (Fig 2; S1 Table). Five miRNA seeds were homologous to miRNAs in the Rfam [35] or miRBase [36] databases, namely: mir-398 (Rfam ID: RF00695), mir-450 (RF00708), mir-598 (RF01059), mir-785 (RF02244) and cte-mir-981 (miRBase ID: MI0010092). Reads representing miRNAs accounted for 16% and 9% of all sRNA reads sequenced from male and female adults of *S*. *haematobium*, respectively. The most highly transcribed miRNAs in male worms were sha-mir-1 (5.9% of mapped sRNA reads), sha-mir-71a (5.8%), sha-mir-125b (1.6%), sha-mir-7a (1.0%) and sha-let-7 (0.6%). In female worms, the most highly transcribed miRNAs were sha-mir-71a (3.6% of mapped sRNA reads), sha-mir-1 (2.0%), sha-mir-71b (0.7%), sha-mir-125b (0.7%) and sha-bantam (0.3%) (S1 Table). In total, 59 of 89 miRNAs (66%) exhibited substantial female-biased transcription, with CPMs of more than twice that of respective miRNAs in the male library. Of these 59 miRNAs, 44 (75%) were novel (or ‘orphan’) miRNAs without known homologs in other schistosome species studied to date or in miRNA databases. In contrast, five of the 89 miRNAs (6%) had male-biased transcription (more than two-fold higher CPM values), with four of them being orphan miRNAs (Fig 2; S1 Table).

A total of 798 potential binding interactions were identified between *S*. *haematobium* miRNAs and predicted 3’-UTRs, of which 519 represented unique interactions between 46 miRNAs and 332 genes (excluding isoforms; S2 Table). Genes with more than five miRNAs predicted to bind a 3’-UTR included a “methyltransferase-like protein 14” (MS3_05841; eight miRNAs), a “serine/threonine-protein phosphatase 2B” (MS3_00821; seven miRNAs), a “putative member of the *sno* and *ski* oncogene family” (MS3_02059; six miRNAs) and an uncharacterised protein (MS3_10507; six miRNAs) (S2 Table). The majority (*n* = 38; 82.6%) of miRNAs predicted to bind 3’-UTR elements were associated with more than one gene; these miRNAs included sha-miR-450_RF00708 (104 genes), sha-miR-71a (53 genes), sha-miR-71b (43 genes), sha-miR-new_38 (39 genes) and sha-miR-new_36 (37 genes).

### Other sncRNAs of *S*. *haematobium*

A large number of sRNA reads mapped to rRNA (22,697,561 of 56,793,790 total reads mapped; 40%; 747 clusters) and tRNA (3,386,888 of 56,793,790 total reads mapped; 6%; 782 clusters) regions (S3 and S4 Tables). Of the remaining reads, which formed 3875 sncRNA clusters, 49 ShortStack precursors had RNA products of a consistent size and characteristic, containing a representative RNA sequence with conservation to sRNA in the Rfam database (S5 and S6 Tables). Among these precursors were clusters encoding most known spliceosomal small nuclear RNA (snRNA) classes, including ten U6 (RF00026), three U5 (RF00020), three U1 (RF00003), two U6atac (RF00619), two U2 (RF00004) and one U4atac (RF00618) sequences. Of these snRNAs, U1 and U2 were the most highly transcribed (CPM for U1 in library of male worms: 91.9; CPM for U2 in library of female worms: 74.4). Three other clusters encoded the small nucleolar RNAs (snoRNAs) SNORD15 (RF00067; two clusters; CPM range in library of male worms: 66.7–67.8; library of female worms: 56.3–58.8) and SNORA19 (RF00413; one cluster; CPM range: 0.7–0.8) (S5 Table).

Additionally, 16 elements had similarity to “hammerhead 1 ribozyme-like RNA” (RF00163; HHR) within sRNA clusters that were between 55 and 404 nucleotides in length, of which seven and two overlapped completely with DNA predicted to encode SINE (short interspersed nuclear element)/tRNA and LINE (long interspersed nuclear element)/RTE-BovB elements, respectively (S5 Table). None of these clusters overlapped with a region in the genome predicted to encode tRNAs (to which repetitive elements coding for HHRs can be similar in sequence [42]), suggesting that they are *bona fide* SINEs/LINEs. Of all 16 clusters, nine were predicted (using ShortStack) to be ‘hairpin-derived’. Eleven sRNA cluster sequences shared 95–100% sequence identity with predicted *S*. *haematobium* HHRs in the Rfam database. In addition, one cluster (Cluster_22636) matched (91% nucleotide identity across 56nt using blastn [43]) to a previously characterised *S*. *haematobium* HHR (accession number: AF036390.1 [42]). Matching regions of the consensus sequences of these clusters were extracted and, using RNAfold, folds resembling that of an HHR were predicted for eight sequences. Three of the 11 putative HHRs derived from sRNA clusters (Cluster_12997, Cluster_13427 and Cluster_13865) contained the CUGANGA motif, which is a conserved part of the HHR catalytic core [42]. All HHR-like clusters were transcribed at low levels, except for Cluster_16211 and Cluster_86205 which were predominantly transcribed at higher levels in libraries representing male (CPM: 13.2) and female (CPM: 16.5) adults of *S*. *haematobium*, respectively.

In addition to snRNAs and hammerhead-like RNAs, 30 potential miRNA precursors were identified, including mir-80 (RF00817) and lin-4 (RF00052), which lacked the required characteristics of miRNAs, including not being identified by miRDeep2 and not being predicted to have the characteristic miRNA hairpin secondary structure (S5 Table).

### ‘Orphan’ sRNA elements

**Fig 3:**
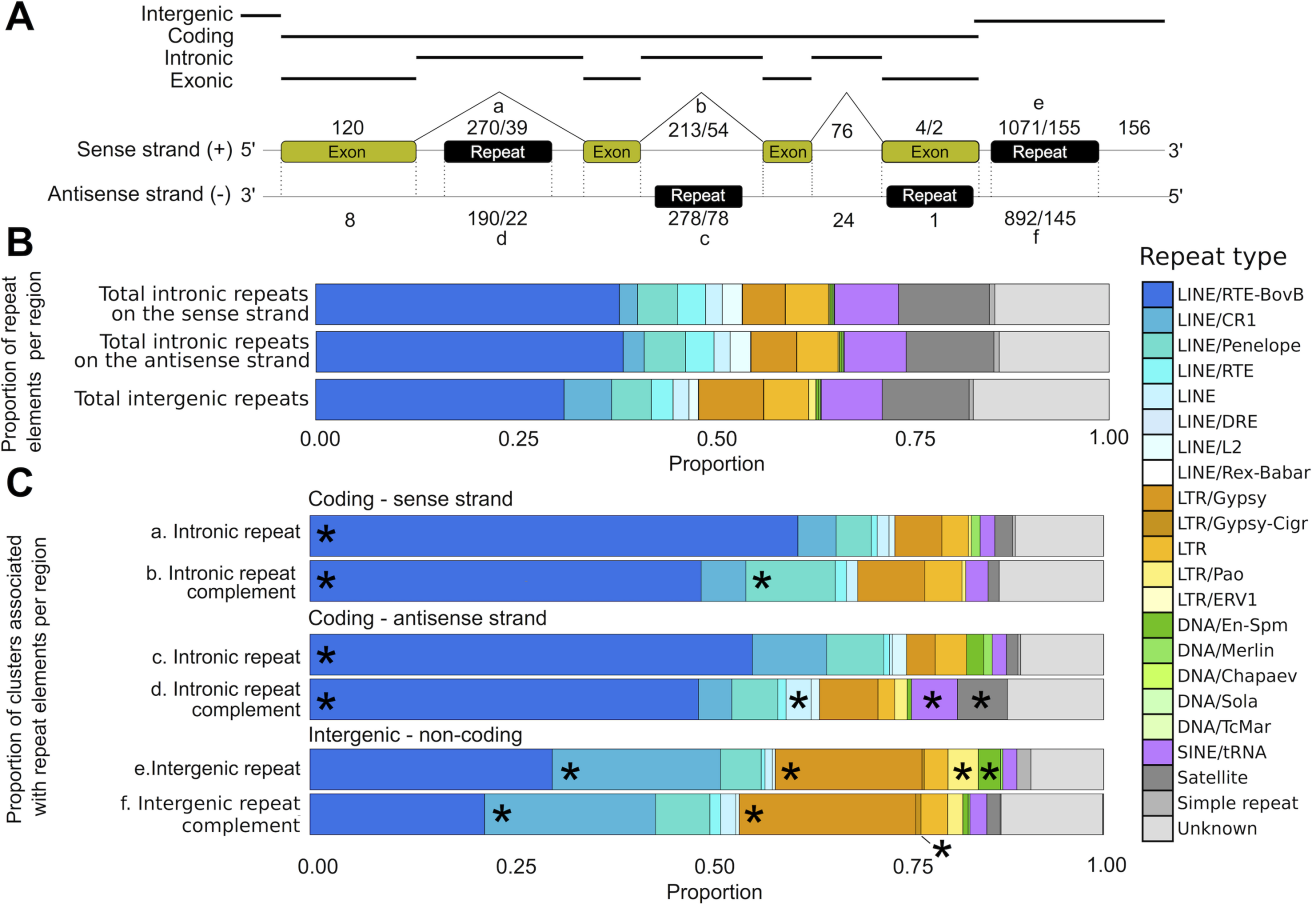
Small RNA clusters within the genome of *Schistosoma haematobium*. (A) Small RNA clusters were identified in intergenic, intronic and exonic regions of the genome. Numbers represent mapped, complete/partial small RNA clusters in these regions on the sense and antisense strands, respectively. (B) Proportion of annotated repeat elements within the genome. (C) Proportion of clusters identified within annotated repeat elements of the genome. Clusters enriched within specific repeat families (one-sided Fisher’s exact test, *p* < 0.05) are marked with an asterisk. LINE, long interspersed nuclear element; LTR, long terminal repeat; SINE, short interspersed nuclear element.

In addition to miRNAs and other known sncRNAs, 3798 sRNA clusters in the genome of *S*. *haematobium* were predicted to share characteristics of non-randomly degraded RNA but could not be annotated based on known sRNA elements in other organisms. To explore these elements in more detail, we assessed whether these sRNA clusters were represented in coding or non-coding regions of the genome and whether they overlapped with a repeat region (S6 Table and Fig 3A). In total, 120 clusters were within exons (from 102 genes), of which 25 were within the translation start site (TSSa) of the gene. Of these 25, two clusters had high transcription levels in the library derived from male worms (CPM range 12.1–43.1), whereas the two most highly transcribed sRNAs in the library derived from female worms exhibited moderate transcription levels (CPM range: 6.6–8.6). In coding regions, eight clusters of sRNAs were on the strand complementary to an exon and were thus predicted to play a role in (siRNA-mediated) gene silencing (Fig 3A). Of these clusters, Cluster_26190, Cluster_8504 and Cluster_84791 were the most highly transcribed in one or both sexes (CPM range: 1.2–24.1). On the sense strand, 483 clusters were found within introns, of which 270 and 213 clusters mapped to regions containing repeat elements on the sense and antisense strand, respectively (Fig 3A and 3B; S6 Table).

Approximately 1% of the reads representing uncharacterised sRNA clusters mapped to transposable elements (TEs; such as DNA/En-Spm, DNA/Merlin, LINEs, long terminal repeats (LTRs) and SINEs/tRNAs; Fig 3A, 3B and 3C) with a slight antisense bias (> 51% of reads), consistent with Piwi-interacting RNA-like elements (piRNAs) [44]. Although schistosomes lack a canonical Piwi pathway [15, 16], three sRNA precursors complementary to repeat elements were identified in the genome. However, these elements were not highly transcribed in libraries derived from male or female worms (CPM range: 0.4–2.3; S6 Table).

## Discussion

Employing a substantially improved gene set for *S*. *haematobium* with enhanced gene annotation (relating to 3’- and 5’-UTRs, longer coding sequences and reduced redundancy) and sRNA-Seq libraries for male and female adults of *S*. *haematobium*, we defined here the transcribed complements of miRNAs and other sncRNAs in this species. Although the number of miRNAs transcribed in *S*. *haematobium* adults was comparable with numbers reported for other schistosome species studied thus far [10, 11, 13, 21], there was little conservation of miRNA homologs among schistosome species, and many miRNAs in *S*. *haematobium* appeared to be species-specific. These findings are consistent with reports of a substantial loss of ‘conserved’ miRNAs from flatworms and a gain of novel miRNA families [8, 10]. For example, the 12 schistosome-specific miRNAs conserved between *S*. *mansoni* and *S*. *japonicum* [10] were not found in *S*. *haematobium*, and of the miRNAs reported as *S*. *mansoni*-specific [10], only one was conserved and shared by *S*. *haematobium*. In this context, it is noteworthy that only miRNAs transcribed in adult *S*. *haematobium* worms were identified in this study. Thus, the apparent lack of some miRNA homologs might be explained by their specific transcription in one or more developmental stages for which data exist for *S*. *mansoni* and/or *S*. *japonicum*, but not yet for *S*. *haematobium*.

For three other miRNAs—mir-190, mir-281 and mir-8451—reported to be conserved among the Bilateria, Protostomia and Platyhelminthes, respectively [10], no representatives were detected in *S*. *haematobium*. For mir-8451, this finding might be explained by this miRNA not being transcribed in the adult stages of schistosomes, consistent with earlier findings [10, 13, 21]. However, transcription in adult stages has been reported for mir-190 in both *S*. *japonicum* [13, 21] and *S*. *mansoni* [10], and for mir-281 in *S*. *mansoni* [10], suggesting that homologs of these miRNAs were not identified here in *S*. *haematobium* due to the stringency with which miRDeep2 defines high-confidence homologs, requiring an exact nucleotide match for positions 2–8 (‘seed’) of the mature sequence. To test this hypothesis, we assessed whether mature and precursor sequences of sma-mir-190, sma-mir-281 and sma-mir-8451 were homologous to any *S*. *haematobium* miRNAs identified using a less stringent (blastn -task “blastn-short”) approach [43]. This analysis revealed exact matches of 7–9 nucleotides to several *S*. *haematobium* miRNAs (including novel or orphan sequences) for all three *S*. *mansoni* sequences. However, when directly comparing positions 2–8 of the homologous mature sequences of *S*. *haematobium* and *S*. *mansoni*, exact matches could not be inferred for any of them. This finding suggests that the lack of homologs for some *S*. *haematobium* miRNAs is due to different approaches used to infer consensus pre-miRNA sequences and mature sequences from mapped sRNA reads. Thus, sequences were not annotated as homologs to a known sequence, unless homology could be inferred using the stringent, high-confidence approach employed in miRDeep2.

Future studies should focus on defining miRNA complements of additional developmental stages of *S*. *haematobium* to allow for a better comparison to miRNAs reported in different developmental stages of other schistosome species [13, 21], and to assess potential losses/gains of particular miRNAs in *S*. *haematobium* and other species of schistosomes. The availability and quality of genome assemblies can also have a marked impact on miRNA detection, as exemplified in a recent publication [7], which demonstrated significantly improved inference of miRNA employing a draft genome for *Fasciola hepatica* over findings without a genome [45]. Thus, additional miRNAs are likely to be detected using enhanced genome assemblies for *S*. *haematobium* in the future.

The present finding that many miRNAs of *S*. *haematobium* are primarily transcribed in female adult worms is largely concordant with those reported previously for *S*. *mansoni* and/or *S*. *japonicum* [10, 21]. Specifically, female-biased transcription of six and two miRNAs, whose homologs also have female-biased transcription in *S*. *mansoni* [10] and *S*. *japonicum* [21], respectively, and male-biased transcription for mir-1b (reported for *S*. *mansoni* [10]) were established. Notably, among the female-biased transcripts were the three miRNAs, sha-mir-71b, sha-mir-2b and sha-mir-2c (encoded in a cluster on scaffold KL251164.1), whereas the related miRNAs sha-mir-71a and sha-mir-2a (encoded in a cluster on scaffold KL250488.1) did not show sex-biased transcription. These results are consistent with reports of a mir-71/mir-2 cluster duplication in *S*. *mansoni* [10, 11] and *S*. *japonicum* [14, 34] on the female-sex (W) chromosome and on one autosome (chromosome 5), and further support roles in sex-specific traits, sexual differentiation, pairing of adult worms and reproductive processes in schistosomes.

Similar to the finding of conserved miRNAs among schistosomes, female-biased transcription for the majority of novel, *S*. *haematobium*-specific miRNAs, supports proposed roles in reproductive biology and/or pairing of adult worms. Moreover, the four novel miRNAs transcribed principally in male adults, including one miRNA (sha-miR-new_46) encoded on the same scaffold as the mir-71/mir-2 cluster (KL250488.1) with a CPM more than 20-times higher than that in the library derived from adult female worms, suggest male-specific roles for these miRNAs in gene regulation.

In addition to the analysis of transcriptional profiles, an improved gene annotation and inference of 3’-UTRs enabled a homology-based prediction of miRNA binding sites in the genome. Predictions of targets of *S*. *haematobium* miRNAs (S2 Table) did not agree with those made for the respective *S*. *mansoni* homologs [11]. The number of targets for mir-71a and mir-71b identified here was similar to that of a previous study of *S*. *mansoni* miRNAs [11], but overall, the number and identity of targets were discordant with results from the present study. Reasons for this discrepancy might be: (i) that the previous study of *S*. *mansoni* employed miRanda (minimum score threshold: 120), whereas here, a combination of miRanda (minimum score threshold: 300) and PITA was used to predict targets; or (ii) due to difference in the number of annotated 3’-UTRs in the gene sets between the two species. The current *S*. *mansoni* gene set (PRJEA36577; WormBase ParaSite v.9) has annotations for 3’-UTRs representing 4,534 transcripts, in contrast to 6,696 transcripts with 3’-UTRs identified for *S*. *haematobium*. A comparison of 3’-UTRs of 10,015 pairwise (amino acid) orthologs between *S*. *haematobium* and *S*. *mansoni* showed that only 21% (*n* = 2064) of orthologs had a 3’-UTR annotation in both species. Clearly, future work (including additional RNA-Seq libraries and/or HITS-CLIP [46]) to unify and improve the annotation of UTRs in schistosome species is warranted to gain a better understanding of miRNA-targeting in schistosomes. In this context, it would also be valuable to experimentally identify and validate miRNA targets, as has been recently reported for *S*. *japonicum* [47].

Other sRNAs identified included snRNAs, snoRNAs, HHR-like RNAs and uncharacterised sncRNAs, some of which were predicted to be TE-derived. For snRNAs, most classes known to constitute the spliceosome and the minor spliceosome [48] were identified, but representatives of U4, U11 or U12 were not. It is likely that *S*. *haematobium* sequences representing these three classes are more similar in sequence to the Rfam models for U4atac, U1 and U2, respectively, and were thus assigned to the latter (or other) classes. This prediction is supported in that two to ten loci were identified to encode five different classes of snRNAs. Similarly, only three representatives of snoRNAs which are predicted to function in the biogenesis of other rRNAs, tRNAs and snRNAs [49] were detected. Other snoRNAs in *S*. *haematobium* might not have been classified due to their sequence divergences from Rfam models and might thus be amongst the 3798 unclassified sRNAs.

Of the unclassified sRNAs, sRNA products of a consistent size mapped to the antisense strand of predicted TEs. Although *S*. *haematobium* and other schistosomes lack a canonical Piwi pathway [15, 16], this information suggests that schistosomes can suppress TEs via TE-derived sncRNAs, thus contributing to genomic stability, as proposed for sncRNAs in *S*. *japonicum* [13]. Schistosomes lack Piwi and Vasa proteins and other argonaute proteins of the Piwi family [15], which have been recognised as being relatively conserved among members of the Deuterostomia and Ecdysozoa [15]. Thus, it is plausible that *S*. *haematobium* and other schistosomes have evolved alternative sRNA-mediated RNA degradation pathways to control ‘jumping’ genes [50], similar to those in nematodes [51].

It is possible that the degradation of TEs is regulated by some of the 16 HHR-like sRNAs identified here, with varying levels of evidence and confidence (i.e. having a hairpin-fold, conserved motifs and/or sequence homology; S5 Table). HHRs are RNA enzymes found within repetitive TEs, such as SINEs [52]. They can self-cleave the SINE in which they are encoded, thus controlling the propagation of TEs in the genome. Additionally, some HHRs can cleave other RNA targets [42], thus regulating their transcription, and might also have roles in the processing of tRNAs, sRNAs, and RNAi inhibition [52]. Although the present results do not allow for the prediction of a function for HHRs identified in *S*. *haematobium*, they provide experimental evidence (at the RNA level) that they are encoded in the genome. Combined with earlier reports of both HHRs [52] and TEs [53] in several trematodes, the present data provide a foundation for future investigations of these intriguing RNA molecules and their roles in the molecular biology of schistosomes.

## Conclusions

The present work represents the first genome-wide miRNA and sncRNA resource for *S*. *haematobium*, extending previous work on schistosomes, and providing additional evidence with regard to the conservation of miRNAs across flatworms [8]. The outcomes from this work should facilitate future research of the post-transcriptional regulation of genes in schistosomes, and the roles that sncRNAs play in development and parasite-host interactions. Given the proposed involvement of these RNAs in the infection process, parasite-host cross-talk and development of drug resistance, and their potential relevance as drug targets [46, 54], an improved understanding of sncRNAs transcribed in developmental stages within the definitive host, are much needed. The findings from the present study provide a foundation for such future endeavours.

## Supporting information

S1 Table Predicted miRNA based on mapping of small RNA libraries to the *S*. *haematobium* reference genome. (XLSX)

S2 Table Predicted miRNA target genes, their associated transcription levels (in transcripts per million, TPM) and annotation. (XLSX)

S3 Table rRNA-like clusters of mapped sRNA reads in the genome of *S*. *haematobium*. (XLSX)

S4 Table tRNA-like clusters of mapped sRNA reads in the genome of *S*. *haematobium*. (XLSX)

S5 Table Classified sncRNA clusters in the genome of *S*. *haematobium*. (XLSX)

S6 Table Unclassified sncRNA clusters in the genome of *S*. *haematobium*. (XLSX)
